# Quantifying Beetle-Mediated Effects on Gas Fluxes from Dung Pats

**DOI:** 10.1371/journal.pone.0071454

**Published:** 2013-08-07

**Authors:** Atte Penttilä, Eleanor M. Slade, Asko Simojoki, Terhi Riutta, Kari Minkkinen, Tomas Roslin

**Affiliations:** 1 Department of Agricultural Sciences, University of Helsinki, Helsinki, Finland; 2 Department of Zoology, University of Oxford, Oxford, United Kingdom; 3 Department of Food and Environmental Sciences, University of Helsinki, Helsinki, Finland; 4 Environmental Change Institute, University of Oxford, Oxford, United Kingdom; 5 Department of Forest Sciences, University of Helsinki, Helsinki, Finland; University College London, United Kingdom

## Abstract

Agriculture is one of the largest contributors of the anthropogenic greenhouse gases (GHGs) responsible for global warming. Measurements of gas fluxes from dung pats suggest that dung is a source of GHGs, but whether these emissions are modified by arthropods has not been studied. A closed chamber system was used to measure the fluxes of carbon dioxide (CO_2_), methane (CH_4_) and nitrous oxide (N_2_O) from dung pats with and without dung beetles on a grass sward. The presence of dung beetles significantly affected the fluxes of GHGs from dung pats. Most importantly, fresh dung pats emitted higher amounts of CO_2_ and lower amounts of CH_4_ per day in the presence than absence of beetles. Emissions of N_2_O showed a distinct peak three weeks after the start of the experiment – a pattern detected only in the presence of beetles. When summed over the main grazing season (June–July), total emissions of CH_4_ proved significantly lower, and total emissions of N_2_O significantly higher in the presence than absence of beetles. While clearly conditional on the experimental conditions, the patterns observed here reveal a potential impact of dung beetles on gas fluxes realized at a small spatial scale, and thereby suggest that arthropods may have an overall effect on gas fluxes from agriculture. Dissecting the exact mechanisms behind these effects, mapping out the range of conditions under which they occur, and quantifying effect sizes under variable environmental conditions emerge as key priorities for further research.

## Introduction

Climate change is now one of the greatest drivers of environmental modification worldwide [1], with agriculture and food production being major sources of the greenhouse gases (GHGs) responsible for global warming [2], [3]. Of all anthropogenic GHG emissions, 18% are produced by cattle farming – of anthropogenic nitrous oxide (N_2_O) and methane (CH_4_) emissions, the corresponding figures are 65% and 35–50%, respectively [2]. With global increases in meat consumption, the reduction of GHG emissions from livestock production has become of great importance [3]. Dung pats left on fields are a known source of both CH_4_ and carbon dioxide (CO_2_) [4], [5]. Hence, processes contributing to the decomposition of dung, and to associated gas fluxes, are of key interest in assessing gas fluxes from cattle farming.

The decomposition of dung is primarily a biotic process involving a range of organisms [6]–[8]. Yet, most studies on GHG fluxes from dung consider the impacts of abiotic conditions, such as temperature and moisture (e.g. [9], [10]), or of plant and soil interactions [11], [12], whereas the role of the dung fauna has received considerably less attention. While recent findings suggest that the activity of earthworms may increase emissions of N_2_O and CO_2_ from dung pats [13], [14], and increase the rate of CH_4_ production in soils [15], [16], the potential for other invertebrates to modify emissions of GHGs is little explored (but see [17]–[19]).

In temperate agricultural grasslands, dung beetles (Coleoptera: Scarabaeoidea) are the most important invertebrate contributors to dung decomposition [7]. Although the overall ecosystem services provided by dung beetles have gained much interest (see [20] for a review), their impact on GHG emissions has received insufficient attention. There is some evidence that they may influence nitrogen fluxes; beetles tunneling below dung pats have been found to reduce the volatilization of ammonia (NH_3_), thus improving the availability of inorganic nitrogen in the soil [5], [17], [21]. However, the effect of invertebrates on CH_4_ fluxes from dung has not been studied. As CH_4_ is formed in anaerobic conditions [22], and as dung beetles aerate the dung [23], we suggest that they may play an important role in reducing emissions of CH_4_ from dung. Furthermore, some scarab beetles have also been shown to harbor intestinal methanogens, suggesting another possible mode for dung beetles to influence CH_4_ fluxes [24], [25].

In this study, we empirically quantify the impact of dung beetles on the fluxes of three major GHGs: nitrous oxide (N_2_O), carbon dioxide (CO_2_), and methane (CH_4_) from dung pats. *A priori*, we hypothesized that the aerating influence of dung beetles would increase fluxes of N_2_O and CO_2_ but decrease fluxes of CH_4_. To offer a first assessment of whether dung beetles may contribute to mitigating or accelerating climate change, we then quantified the cumulative effect of dung beetles on net releases of GHGs from dung pats exposed under semi-natural conditions over the main growing season (June–July) of the boreal zone. Overall, we describe a clear-cut signature of dung beetles on local GHG fluxes under the present experimental conditions, thus offering a seminal suggestion that dung beetles may exert a wider impact on GHG fluxes from agriculture.

## Materials and Methods

### Experimental Design

To examine the effect of dung beetles on gas fluxes from dung pats, we constructed three treatments: 1) dung pats with dung beetles (n = 10), 2) dung pats without dung beetles (n = 10), and 3) controls with neither dung pats nor dung beetles (n = 2). As the latter treatment will capture gas fluxes from the soil of the pasture, it can be used as a point of comparison when evaluating gas fluxes from dung *per se*.

**Figure 1:**
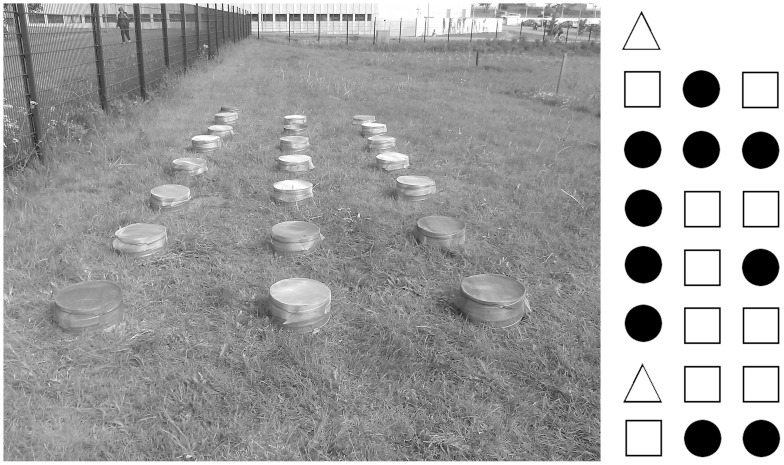
Experimental design used in measuring gas fluxes. (A) Twenty-two mesocosms were placed in an agricultural field, separated by distances of 70 cm. (B) These mesocosms were randomly assigned to three different treatments: 1) dung with dung beetles (open squares; n = 10); 2) dung without dung beetles (filled circles; n = 10), and 3) chambers containing neither dung nor beetles (open triangles; n = 2).

All treatments were implemented on a grass sward reflecting a multiannual Finnish pasture. The experimental area (located in Viikki, Helsinki, Southern Finland; 60° 13′ 31′′ N 25° 1′ 0′′ E; Fig. 1) is owned by the University of Helsinki, and hence no specific permission was required for this locality. Within the experimental field, the spatial distribution of replicates within each treatment was randomized among a set of 22 mesocosms (Fig. 1). Each mesocosm was constructed from a 25 cm section of 0.5 mm-thick air duct pipe of zinc-coated steel (∅ 31.5 cm). Each pipe section was then installed 10 cm into the ground, leaving a 15 cm-high collar above ground (Fig. 1).

Dung beetles were collected in pastures of the Koskis Manor in Salo, Southwestern Finland (60° 22′ 49′′ N 23° 17′ 39′′ E) on May 31^st^ and June 1^st^ 2011. Explicit permission for sampling was obtained from the owners of the Manor, Fredrik and Helena Von Limburg Stirum. The dung beetles collected were all in the genus *Aphodius* – the dominant group of dung beetles inhabiting Northern European pastures [26]. These species are small and typically live within the dung or at the soil-dung interface (endocoprids), although one species (*A. erraticus*) tunnels below the dung pat (paracoprid). The beetles were kept in moist paper at +4°C until being used in the experiment. No protected species were sampled.

Dung for the experiment was gathered from the barn at the Viikki Study and Research Farm, owned by the University of Helsinki. Explicit permission was obtained from the Director of the farm, Miika Kahelin. The dung was collected from a herd of some twenty heads of Ayrshire cattle, all adult dairy cows. At the time of dung collection, the cattle had been grazing daily for approximately a month on improved pastures sown with a mix of timothy (*Phleum pratense*) and meadow fescue (*Festuca pratensis*) with a smaller component of red clover (*Trifolium pratense*). Outdoor grazing time ranged from 4 to 5 hours per day between 8 AM and 2 PM, with the dung collected as the cattle entered the barn for within-stall milking. When indoors, the cattle was fed additional silage *ad lib*, a standard concentrate (Maituri 20 and Amino-maituri 30, Raisio Oyj, Raisio, Finland) and magnesium-selenium-minerals (Pihatto-Melli; Raisio Oyj, Raisio, Finland). No animal in the heard had been given antibiotics or antiparasitic treatments. All dung was manually homogenized before partitioning into experimental pats.

On June 7^th^, the dung was split into pats of 1.2 litres each, and distributed into the mesocosms. The pat size used was based on two criteria: first, while natural cow pats will vary in size, we used a size within the typical size range [27], second, we chose a size that left a *ca.* 5 cm rim of vegetation outside of the pat within the mesocosm, mimicking the situation on a natural pasture. Seven species of beetles were then distributed among the mesocosms in numbers reflecting their natural distribution in the field (Table 1).

**Table 1:** Dung beetle abundances used in the experiment.

Species	Individual dry mass (mg)	Per chamber*	Total†
*Aphodius ater* (De Geer, 1774)	4.2	73	730
*Aphodius fimetarius* (Linnaeus, 1758)	9.4	5	50
*Aphodius depressus* (Kugelann, 1792)	9.1	4	40
*Aphodius erraticus* (Linnaeus, 1758)	13.6	41	410
*Aphodius haemorrhoidalis* (Linnaeus, 1758)	2.2	11	110
*Aphodius pusillus* (Herbst, 1789)	1.0	7	70
*Aphodius fossor* (Linnaeus, 1758)	26.1	12	120
Total		153	1530

Information on species-specific dry masses taken from [39].

* Species-specific number of individuals added to each replicate chamber in treatment 1.

† Species-specific total counts used in the experiment.

### Chamber Measurements

To evaluate gas fluxes from the dung pats, we used a closed chamber method [28]. The chambers were constructed following the USDA-ARS GRACEnet Chamber-based Trace Gas Flux Measurement protocol 2003 [29], [30]. The sections of air duct pipe used as mesocosms (see above) also formed the chamber collars. Between measurements, these collars were closed by a metal mesh, allowing air circulation while keeping the dung beetles from escaping. The vegetation inside the chambers was kept low by manual trimming. For additional details on chamber design see Appendix S1.

### Gas Flux Measurements

Gas fluxes were measured between 09∶00–17∶00 hours on seven occasions between June 8^th^ and July 27^th^ 2011, corresponding to days 1, 6, 10, 15, 20, 30 and 50 of the experiment (described in Appendix S1). For measurements of CH_4_ and N_2_O gas samples were taken with a syringe after 5, 10, 20, and 30 minutes of the chamber being sealed, and injected into glass vials (3-ml Labco Exetainers® with double septa, Labco Ltd., Buckinghamshire, UK). CH_4_ and N_2_O were then quantified in parts per million (ppm by volume) by gas chromatographs (HP 5890 Series II, Hewlett Packard, Palo Alto, CA, U.S.A.) equipped with thermal conductivity, flame ionization and electron capture detectors.

Measurements of CO_2_ fluxes were carried out in the field with a portable device (a modified version of the SRC-1 soil respiration chamber and the EGM-4 infrared CO2 analyzer, PP Systems, Amesbury, MA, U.S.A.; for similar designs see [28], [31]). CO_2_ fluxes were measured approximately four hours after the CH_4_ and N_2_O samples were taken (between 13∶00 and17∶00 hours). The sampling time for each chamber was set to 80 seconds and the measuring interval was 4.8 seconds. Ambient temperature was recorded during the sampling of all gases, for later scaling of gas fluxes to temperatures. For additional details on gas flux measurements see Appendix S1 and for a description of environmental conditions during the experiment see Appendix S2.

Different greenhouse gases have different Global Warming Potentials (GWP) [1]. To derive a joint measure of the warming effect of the gas fluxes quantified above, we therefore used compound-specific multipliers suggested by the IPCC [1] (25 for CH_4_ and 298 for N_2_O) to weigh together the contribution of individual compounds into the general currency of “CO_2_ equivalents”, at a 100-year time horizon.

A technical problem with the rubber septa used in the sampling vials resulted in the complete loss of data on all CH_4_ and N_2_O fluxes for the first sampling date (June 8, 2011). During the following measurement round (June 14; day 6 of the experiment), heavy rains half-way into the measurements resulted in the loss of CH_4_ and N_2_O readings for two out of ten chambers with beetles and four out of ten chambers without beetles.

To replace the lost measurements of CH_4_ and N_2_O fluxes for the first sampling date of 2011, a supplementary experiment was conducted in June 2012. In brief, dung fluxes were measured over three days from fresh dung with and without beetles. A detailed description of and justification for using data from this experiment is given in Appendix S1. In further analyses of cumulative gas emissions from dung pats (see Discussion), estimates of early CH_4_ and N_2_O fluxes of 2011 were hence replaced by estimates from 2012. Estimates of date-specific flux rates (see below, Statistical analyses and Results) were still focused on data for 2011 alone, thus conservatively preventing any influence of experiment-to-experiment variation.

### Statistical Analyses

To analyse how the fluxes of different compounds varied with time and treatment, we used generalized linear mixed-effects models (GLMMs). A separate, compound-specific model was built for each response (i.e. for fluxes of CO_2_, CH_4,_ N_2_O and CO_2_ equivalents, respectively). The models were fitted in SAS v. 9.2, procedure mixed (SAS Institute Inc., Cary, NC), using a repeated-measures structure with chamber as the subject. To account for the non-independence of consecutive measurements, we assumed a first-order antedependence structure. To allow the strength of dependence to vary with the specific pair of measurements being referenced we specified TYPE = ANTE(1) [32].

To evaluate the statistical significance of the patterns observed, we used the fitted model to derive 95% confidence limits for each mean. Flux estimates with confidence intervals excluding zero were then interpreted as statistically significant sources (CL>0) or sinks (CL<0).

## Results

### Temporal Patterns in Gas Fluxes

A clear imprint of dung beetles was evident on all gas fluxes examined.

#### Fluxes of CO_2_

**Figure 2:**
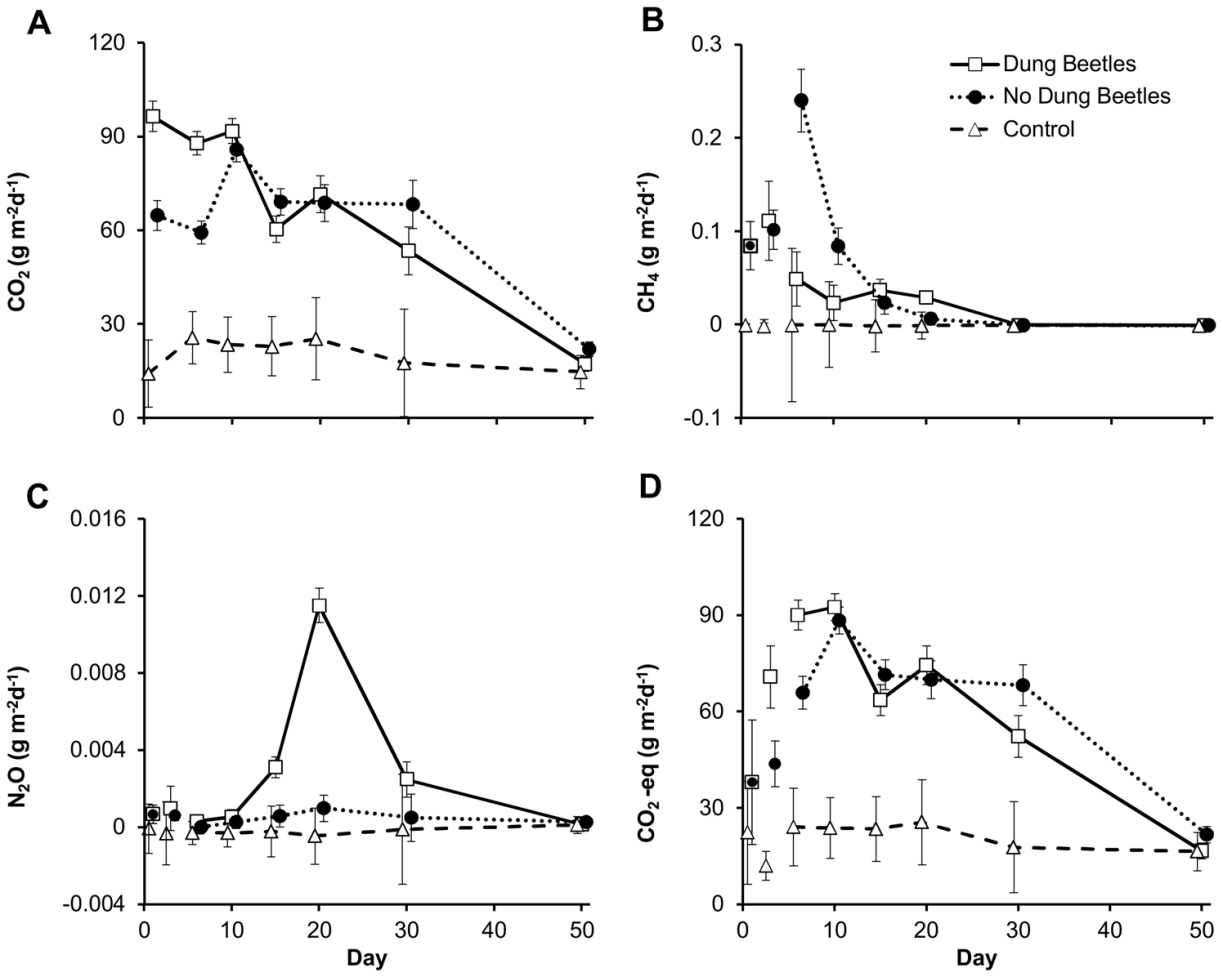
Fluxes of (a) CO_2_ (b) CH_4_ (c) N_2_O and (d) carbon dioxide equivalents. Light gray symbols refer to empirical observations, with treatments identified by the same symbol styles as used in Fig. 1. Symbols with 95% confidence limits show least squares means estimated by a GLMM model (for details, see text and Table 2). To reveal overlapping data points, empirical values were slightly offset in the horizontal dimension. As measurements of CH_4_ (panel b) and N_2_O (panel c) were lost for the first measuring date of 2011, these values are replaced by estimates from a separate experiment conducted in 2012 (see Appendix A for details). For clarity, estimates of 2011 are connected by lines, whereas estimates from 2012 are shown as separate data points (referring to arithmetic means with confidence limits derived from a *t*-distribution). Note the different scales of the y-axes, and that treatments are identified by the same symbols as in Figure 1.

Emissions of CO_2_ differed significantly among treatments (Table 2). Overall, fluxes from the control treatment (containing neither dung nor beetles) were significantly lower than fluxes from treatments with dung (Fig. 2a). In the two treatments with dung, emissions peaked markedly earlier when dung beetles were present than when they were absent (on the first versus 10^th^ day of the experiment; Fig. 2a).

**Table 2:** Generalized linear mixed-effect models of changes in fluxes over time.

	Effect	F Value^a^	Num DF	Den DF	P value
*CO_2_*					
	Treatment^b^	95.03	2	23.5	<.0001
	Measurement^c^	138.44	6	31.4	<.0001
	Measurement×Treatment	40.72	12	42.2	<.0001
*CH_4_*					
	Treatment	28.60	2	21.3	<.0001
	Measurement^d^	11.29	5	33.7	<.0001
	Measurement×Treatment	12.91	10	41.1	<.0001
*N_2_O*					
	Treatment	75.05	2	33.3	<.0001
	Measurement^d^	38.49	5	29.9	<.0001
	Measurement×Treatment	42.09	10	33.2	<.0001
*CO_2_ equivalents*					
	Treatment	74.50	2	27.2	<.0001
	Measurement^d^	183.44	5	37.8	<.0001
	Measurement×Treatment	32.43	10	44.5	<.0001

a Type 3 *F*-tests of fixed effects are given.

b Mesocosms with 1) dung pats and dung beetles, 2) dung pats and no dung beetles, or 3) neither dung pats nor dung beetles.

c Measurement day 1, 6, 10, 15, 20, 30 and 50.

d Measurement day 6, 10, 15, 20, 30 and 50.

#### Fluxes of CH_4_

Fluxes of methane differed significantly among treatments (Table 2). In particular, the treatment without dung beetles emitted five times higher amounts of CH_4_ on day 6 of the experiment than did the other treatments (Figure 2b). For the control treatment, fluxes remained minor throughout the experiment. Towards the end of the experiment, the CH_4_ emissions leveled out across treatments. At this stage, all fluxes were close to zero (Fig. 2b).

#### Fluxes of N_2_O

Fluxes of nitrous oxide were relatively low over time (Fig. 2c), but differed among treatments (Table 2). The most pronounced difference among treatments occurred as a distinct spike in N_2_O emissions from the dung pats with dung beetles at day 20 (Fig. 2c). Again, fluxes from the control remained negligible over the full course of the experiment (Fig. 2c).

#### Fluxes of CO_2_ equivalents

Fluxes of carbon dioxide equivalents differed significantly among treatments (Table 2), with the main differences occurring between the control and the two treatments with dung (Fig. 2d). Nonetheless, dung treatments with and without beetles differed significantly from each other on days 6 and 30 of the experiment (Fig. 2d), albeit in different directions. As the absolute fluxes of CO_2_ were much higher than fluxes of CH_4_ and N_2_O (see the scale of ordinate Figs 2a–2c), overall fluxes of CO_2_ equivalents were dominated by the CO_2_ component (compare Fig. 2a vs. 2d).

## Discussion

This study is, to our knowledge, the first to explore the effects of arthropods on GHG fluxes from dung pats. Overall, flux rates from dung were found to be substantial, with fresh dung pats emitting higher amounts of CO_2_ and lower amounts of CH_4_ in the presence of dung beetles. Three weeks after the start of the experiment, emissions of N_2_O showed a distinct peak in the presence of beetles – a pattern not detected in the treatments without dung beetles. Overall, these findings reveal a potential impact of dung beetles on gas fluxes realized at a small spatial scale. While here observed for a specific set of conditions in a specific experimental setting, the current results suggest that arthropods may have a general impact on gas fluxes from agriculture – a prediction which may now be tested by further work.

### Dung Pats Release Large Amounts of Greenhouse Gases

Our study identified dung as a major source of GHGs. Absolute flux rates of CO_2_, CH_4_ and N_2_O in chambers with dung were observed to be high compared to emissions from agricultural soils in general [4], [11], [12], [33] – and compared to the fluxes observed in our control chambers without dung. However, the elevated fluxes from individual pats were of relatively short duration (Fig. 2; see also [4]).

The present results support earlier studies identifying dung as an important source of GHG emissions from agriculture. These studies also found significant fluxes of CO_2_, CH_4_ and N_2_O from dung (e.g. [5], [9], [10], [12], [33]). CH_4_ emissions from the dung of grazing dairy cows have been observed to be particularly high, ranging from 300 to 2040 mg CH_4_ m^−2^ over the first ten days [4]. Even if dung only covers a fraction of the pasture surface, the overall CH_4_ budget of a boreal pasture switches from a CH_4_ sink into a CH_4_ source when emissions from dung pats are taken into account [12]. These considerations highlight the importance of including dung pats, and the factors influencing gas fluxes from them, in studies of agricultural GHG emissions.

### Dung Beetles Modify GHG Fluxes from Fresh Dung

The largest impacts of dung beetles on gas fluxes from dung were found for CH_4_. Initial emissions from six-day old dung pats without beetles were five times higher than emissions from pats with beetles (Fig. 2b). As CH_4_ is formed under anaerobic conditions, the difference between the two treatments can likely be traced to the aerating effect of dung beetle tunnels [23]. By digging holes, beetles may enhance the drying of dung pats and increase the availability of oxygen in the deeper parts of the pats, thus increasing aerobic decomposition, decreasing anaerobic decomposition and reducing methanogenesis. Thus, by oxygenating the dung pat interior, dung beetles may be exerting an effect different from that of earthworms – which are suggested to promote anaerobic decomposition [13], [15].

Dung beetles also significantly modified fluxes of CO_2_, with higher CO_2_ fluxes from pats with beetles during the first week of the experiment. The exact contribution of respiration by the beetles themselves is so far unknown, and should be quantified in further experiments. However, after the 20^th^ day of the experiment, CO_2_ emissions from dung pats lacking dung beetles surpassed those from pats with dung beetles. A similar transient, short-term effect of earthworms on CO_2_ emissions from soil has also been observed [16].

The most unexpected effect of dung beetle presence was a spike in emissions of N_2_O around day 20. Sporadic peaks in N_2_O fluxes have been found before [34], but such patterns are hard to explain, as the formation of N_2_O by microbes is based on complex processes [35]. However, dung beetles have been suggested to increase NO_3_^−^ levels by aerating the substrate, a process leading to more N_2_O being released from denitrification [21]. Similarly, earthworms have been found to increase denitrification [13], possibly by providing optimal conditions for denitrifying bacteria to function in their gut [16], [36]. As a methodological concern, the episodic nature of these pulses also implies that some of them may go undetected.

While our results confirm that dung beetles can significantly modify the temporal patterns of GHG emissions from dung pats, they do not enable us to uncover the exact mechanisms behind them: the current patterns were conditional on the specific circumstances of our experiment (for a description of general environmental conditions, see Appendix S2). Nonetheless, differences in trajectories for individual GHG compounds point to interesting physiochemical processes occurring within the ageing pat, and call for further exploration of causal factors.

### Implications

The fluxes observed in this study allow us to estimate overall GHG emissions over the full course of the experiment by integrating the area under the curves in Fig. 2 (for exact derivations of the following estimates see Appendix S1). Overall, the effects of dung beetles were different on different GHGs. For CO_2_, a change in emission levels between the early and late parts of the experiment (Fig. 2a) caused emission levels to almost converge between treatments (Table 3). Cumulative emissions of N_2_O showed an almost four-fold increase in the presence of beetles (Table 3), whereas for CH_4_, the effects were the opposite: over the course of the experiment, the pooled emissions of CH_4_ from pats with beetles were more than a third lower than those from pats without beetles (Table 3).

**Table 3:** Average cumulative fluxes and CO_2_ equivalents (g m^−2^, ±SD) of greenhouse gases in the different experimental treatments.

Cumulative fluxes^a^				CO_2_ equivalents^b^			
Treatment	CO_2_	CH_4_	N_2_O	CH_4_	N_2_O	CH_4_+ N_2_O	Total
Control (*F*_C_)	986±114	−0.043±0.006	−0.005±0.001	−1.078±0.149	−1.516±0.396	−2.594±0.545	983±114
Dung beetles (*F*_B_)	2924±297	1.071±0.246	0.136±0.037	26.789±6.152	40.380±11.087	67.169±11.528	2991±297
No dung beetles(*F*_N_)	2956±236	1.770±0.376	0.028±0.020	44.237±9.402	8.488±5.680	52.725±11.527	3009±231
*F*_B_ *versus F*_N_ c	*t*_18_ = −0.27,P = 0.79	*t*_18_ = −4.91,P = 0.001	*t*_13.4_ = 8.10^d^,P<0.0001	*t*_18_ = −4.91,P = 0.001	*t*_13.4_ = 8.10^d^,P<0.0001	*t*_18_ = 2.80,P = 0.01	*t*_18_ = −0.15,P = 0.88
(*F*_B_ *-F*_N_)/*F*_N_	−1%	−39%	386%	−39%	386%	27%	−0.6%

a Cumulative fluxes were calculated separately for each chamber as areas under the temporal gas flux curve (Fig. 2; see also Appendix A). For CH_4_, N_2_O and CO_2_ equivalents, measurements from day 1 and 3 were based on a separate experiment conducted in 2012 (see Appendix A), whereas all CO_2_ measurements were based on data collected in 2011.

b Compound-specific multipliers suggested by the IPCC (2007) were used to weigh together the contribution of individual compounds into the general currency of “CO_2_ equivalents”, at a 100-year time horizon. Thus, fluxes of CH_4_ were converted to CO_2_ equivalents through multiplication by a factor of 25, and fluxes of N_2_O through multiplication by a factor of 298. As the net warming impact of carbon first tied by plants, then released from the dung as CO_2_ will differ from that of CH_4_ or N_2_O fluxes from dung (see Discussion), we derive separate subtotals for the cumulative emission of CO_2_ equivalents of CH_4_, N_2_O, and their sum, as well as summing their total (equaling the warming impact of CO_2_, CH_4_, and N_2_O combined).

c Row *F*_B_ *versus F*_N_ shows the results of a compound-specific *t*-test of treatments *F*_B_ (presence of dung beetles) *versus F*_N_ (absence of dung beetles). The last row of the table shows the ratio between fluxes in the presence (*F*_B_) *versus* absence (*F*_N_) of dung beetles as the percentage ((*F*_B_-*F*_N_)/*F*_N_).Variation in degrees of freedom reflects differences between tests based on equal versus unequal variances. (Where not otherwise specified, the test was based on the assumption of equal variances, as supported by a non-significant Levene’s test.).

d Test based on unequal variances (cf. Fig. 2c); test of equality of variances, *F*_9,9_ = 3.81 P = 0.03.

To evaluate the overall warming effect of GHG emissions from dung pats, compound-specific emissions should be gauged against each other. A crucial question is then what currency to use in evaluating the overall effect of dung beetles. In our experiment, emissions of both CH_4_ and N_2_O were dwarfed by fluxes of CO_2_ (Fig. 2). Thus, overall fluxes proved similar across treatments when converted to total CO_2_ equivalents (Table 3), suggesting that the effect of beetles may be negligible. Nonetheless, the beetles had a strong effect on the profile of compounds released (Fig. 1, Table 3). Most crucially, fluxes of CO_2_ may actually offer a secondary concern – as should all carbon taken up as CO_2_ by plants later be released in the same form (i.e. as CO_2_) from dung, then cattle farming might actually be considered to be carbon neutral. Therefore, the main anthropogenic effect is the conversion of some of this carbon to the much more potent GHG of CH_4_, and the concurrent emission of N_2_O. In our experiment, the effect of beetles on overall CH_4_ emissions was strong, with an effect size of more than one-third (−39%; Table 3). Conversely, if CH_4_ and N_2_O fluxes are considered together, then overall, the presence of beetles increased the warming effect of gas fluxes from dung pats by almost a third (+27%; Table 3). However, this increase is due to the specific spike in N2O emissions around day 20, and further experiments are needed to detect whether this a replicable effect of dung beetles *per se*. Calculating the overall warming potential of GHG fluxes from dung – and the effect of beetles thereon – is then no simple exercise, but one urgently needed.

In conclusion, our paper offers a first demonstration that dung beetles can have an impact on GHG fluxes from agriculture. As the patterns reported here could be conditional on specific experimental conditions, they point to some immediate needs for further research. Most urgently, we need to dissect the exact mechanisms behind the patterns observed, map out the range of conditions under which they occur, and quantify the effect sizes under variable environmental conditions. We also note that our study targeted the effects of a specific group of dung beetles (genus *Aphodius*), and that the effects of other beetles of lower abundance but potentially higher functional efficiency (i.e. genus *Geotrupes* [37], [38]) remain to be established. Only by addressing these challenges can we identify the net importance of arthropod-mediated effects on GHG fluxes from dung. While resolving these questions will call for substantial work, we hope that our paper will act as a catalyst for further activity in this field.

## Supporting Information

Appendix S1 Supplementary methods used in measuring gas fluxes.
(DOC)

Appendix S2 Environmental conditions during the experiment.
(DOC)
