# Supplementary material for: Quantifying Beetle-Mediated Effects on Gas Fluxes from Dung Pats
Source: PLoS One. 2013 Aug 7;8(8):e71454. doi: 10.1371/journal.pone.0071454 (PMC3737124; doi:10.1371/journal.pone.0071454)
Supplement: Appendix S1 — Supplementary methods used in measuring gas fluxes. (DOC) [file pone.0071454.s001.doc]

**Appendix S1: Supplementary methods used in measuring gas fluxes**

As a complement to the abbreviated outline of materials and methods given in the paper, we provide here an in-depth description of the exact methods used in the measurement and analysis of the gas fluxes.

*Chamber design*

To evaluate gas fluxes from the dung pats, we used a closed chamber method . This method is based on recording the accumulation of gas content over time within a closed volume of air. If the air is circulating between the chamber headspace and the analyzer (as implemented here for CO_2_), then the method is referred to as a closed dynamic chamber, whereas when samples are taken from a static headspace (as here implemented for CH_4_ and N_2_O), it is called a closed static chamber .

*Measurements of CH_4_ and N_2_O*

To seal off the gas space inside each chamber when taking the gas samples, the chamber collar was covered with an air-tight lid, manufactured from modified air duct cleaning vents (TUTL D 315, Onninen Co., Hyvinkää, Finland). To circulate the air within the chamber once closed, each lid was equipped with a 12V computer fan powered by a 0.8Ah led battery. Once the lid was installed, the volume of the chamber was 10.49 liters. Each lid held two butyl rubber caps each pierced by a piece of Teflon tube (inner Ø 0.96 x outer Ø 1.57 mm) – one for stabilizing air pressure (150 cm) and one for taking gas samples (30 cm). To stabilise pressure while installing the lid onto the chamber collars, one of the butyl rubber caps was removed and then replaced once the lid was in place.

Gas samples were drawn into 20 ml polypropylene syringes through a 3-way stopcock, and then injected directly through a double-wadded septum (VC329) into 3-ml soda glass vials (Exetainer®, Labco Ltd., Buckinghamshire, UK). For accuracy in gas measurements, each vial had been flushed twice with helium and evacuated with a vacuum pump before use.

Gas samples were taken after 5, 10, 20, and 30 minutes of the chamber being sealed. The tubes and syringe were first flushed with 3 ml of the gas and then 9 ml of gas was drawn into the syringe, 1 ml of which was used to flush the injection needle and the rest (8 ml) injected into the 3 ml vial. This procedure created an overpressure in the vial, as needed for successfully injecting the samples into the gas chromatograph. As the overpressure initially used proved excessive, causing some of the septum caps to come loose, the amount of gas injected was changed from 8 ml to 7 ml on June 22^nd^, 2011.

Gas contents of CH_4_ and N_2_O were quantified in parts per million (ppm) with two interconnected HP 5890 Series II gas chromatographs (Hewlett Packard, Palo Alto, CA, U.S.A.) within three weeks (median 5 days) of sampling. The quantification procedure followed Jaakkola & Simojoki , with the exception that helium (He) was used as a carrier gas for both chromatographs and a mixture of Ar (95%) and CH_4_ (5%) as a makeup-gas for the electron capture detector (ECD). One-point calibration for each measured gas was carried out with a certified gas mixture. The nonlinearity of ECD response to N_2_O concentration was corrected by a non-linear empirical function of the measured concentration. An autosampler and a gas sampling valve facilitated the injection of samples into the gas chromatographs.

Temporal drifts of N_2_O readings due to any changes in the sensitivity of ECD during long runs were accounted for by analyzing standard gas samples at 10-sample intervals. The following third-order polynomial equation was fitted to these measurements by using the trendline fitting function of Excel (version 2007, Microsoft Inc., Redmond, WA, U.S.A.), and then used as a multiplier to correct all N_2_O measurements:

$A=\frac{y_{c}}{y_{m}}=ax^{3}+bx^{2}+cx+d$ (1)

where

$A$= correction factor,

$y_{c}$= correct content of standard sample,

$Y_{m}$= measured content of standard sample,

$x$= sample position (sequence number) in the measurement run (1-96),

and $a$, $b$, $c$, and $d$ are fitted parameters.

To estimate the flux of N_2_O and CH_4_ in the experimental chambers, measurements of gas concentrations (c) were regressed on time (t) using the linear fitting algorithm of Excel (version 2007, Microsoft Inc., Redmond, WA, U.S.A.). We then used the slope dc/dt to estimate the overall gas flux, as based on the following equation:

$J=\frac{dc}{dt}*\frac{M}{V_{0}}*\frac{T_{0}}{T}*H$ (2)

where

$J$ = gas flux (g m^-2^ h^-1^)

$\frac{dc}{dt}$ = slope of the linear regression of volumetric gas concentration on time (h^-1^)

$M$ = molar mass of the measured gas (g mol^-1^)

$V_{0}$ = volume at standard condition (m^3^ mol^-1^)

$T_{0}$ = temperature at standard condition (K)

$T$ = temperature (K)

$H$ = chamber height (m)

On June 27^th^, 2011, the N_2_O and CH_4_ fluxes of chamber 19 (with dung beetles) significantly deviated from all other readings of the full experiment. This data point was consequently regarded as an erroneous measurement and omitted from all analyses.

*Measurements of CO_2_*

To measure fluxes of carbon dioxide (CO_2_), the air-tight lid used to seal chambers during measurements was modified to fit the gas probe of a portable device . The probe was installed through the center of the lid and sealed with silicone. To reduce the increase in partial pressure when placing the lid on to the collar before CO_2_-measurements, these lids were made higher than those used for measurements of CH_4_ and N_2_O (see above). Hence, during CO_2_-measurements, the volume of each static chamber was 13.37 liters. To further avoid fluctuations in pressure, a butyl rubber cap was removed while installing the lid onto the chamber, and then replaced.

Estimates of carbon dioxide fluxes were derived from the EGM-4, which automatically records the CO_2_ concentration in the chamber headspace every 4.8 seconds over the 80 second measurement period and fits a linear regression against CO_2_ concentration and time. The fluxes were then converted to g m^-2^d^-1^, using the temperature readings and volume of the chamber.

*Carbon dioxide equivalents*

Different greenhouse gases have different Global Warming Potential (GWP) according to the warming influence of the gas in question . To allow for simpler and more tractable comparisons of compound-specific emissions, multipliers translating emissions of each GHG into CO_2_ equivalents have been estimated by the IPCC (2007). As different gases will remain in the atmosphere for different time spans, these multipliers differ depending on the time horizon considered. The most commonly used time horizon is 100 years, giving CH_4_ and N_2_O multipliers of 25 and 298, respectively (IPCC 2007). To calculate the treatment-specific gas flux for each day of the experiment, we used linear interpolation between the treatment-specific means of sampling dates 1, 6, 10, 15, 20, 30 and 50. To obtain the total amount of CO_2_, N_2_O, CH_4_, and CO_2_ equivalents released through gas fluxes from each treatment, we then summed daily fluxes over the full experiment. Missing readings of CH_4_ and N_2_O (n=2 chambers with beetles and n=4 chambers without beetles; see paper Methods, Gas flux measurements) for day 6 were replaced by the appropriate treatment-and-day-specific means, as was one missing reading for day 10 and one for days 20 (different chambers on each occasion, in both cases a chamber with beetles).

*Supplementary measurements conducted in 2012*

A technical problem resulted in the complete loss of data for CH_4_ and N_2_O fluxes for the first sampling date (June 8) of the main experiment conducted in 2011 (see paper Methods, Gas flux measurements). To replace these lost measurements, a short supplementary experiment was conducted in June 2012.

On 8th June 2012, dung identical to that used in 2011 was split into pats of the same size as used in 2011 (1.2 litres). The dung originated from the same cattle herd as used in 2011, which were fed the same fodder and grazed on the same pasture for the same period of time. No medication had been administered to the cattle in either year. Matching the design of 2011, the pats were split into three treatments: 1) dung pats with dung beetles (n=5; for the specific species composition used in each mesocosm, see Table S1), 2) dung pats without dung beetles (n=3), and 3) controls with neither dung pats nor dung beetles (n=3).

**Table S1. Dung beetle abundances used in the supplementary experiment of 2012.** Information on species-specific dry masses taken from .

| Species | Individual dry mass (mg) | Mesocosm 1* | Mesocosm 2* | Mesocosm 3* | Mesocosm 4* | Mesocosm 5* | Total† |
| --- | --- | --- | --- | --- | --- | --- | --- |
| *Aphodius erraticus* (Linnaeus, 1758) | 13.6 | 30 | 30 | 30 | 20 | 60 | 170 |
| *Aphodius fossor* (Linnaeus, 1758) | 26.1 | 10 | 0 | 0 | 7 | 0 | 17 |
| *Aphodius fimetarius* (Linnaeus, 1758) | 9.4 | 0 | 0 | 6 | 4 | 0 | 10 |
| Total |  | 40 | 30 | 36 | 31 | 60 | 197 |

* Species-specific number of individuals added to each replicate chamber in treatment 1.

† Species-specific total counts used in the experiment.

To measure fluxes of CH_4_ and N_2_O, we used techniques identical to the ones used in 2011, with one exception: instead of using the same chambers as mesocosms and measurement chambers, we used bigger plastic buckets (Ø 58 cm, height 32cm) as mesocosms. From each bucket, the bottom was sawn off, and the resulting mesocosm inserted 20cm into the ground. For flux measurements, we then closed a smaller headspace by inserting a separate closed static chamber into slits cut in the ground. Headspace volume thus remained identical across years. The experiment was conducted in the same experimental fields in Viikki, Helsinki as used in 2011.

Measurements of gas fluxes were conducted on days 1 and 3. As dung beetles were only introduced on day 1, we assumed no differences between treatments at this point, and only measured flux rates in the soil-only versus dung-only treatments. On day 3, flux rates in chambers with and without dung beetles were similar in terms of both CH_4_ (*t*_6_=0.35, P=0.73) and N_2_O (*t*_6_=0.53, P=0.61;see Fig 1 of paper Results).

**References**

1. Alm J, Shurpali NJ, Tuittila E-S, Laurila T, Maljanen M, et al. (2007) Methods for determining emission factors for the use of peat and peatlands flux measurements and modelling. Boreal Environment Research 12: 85-100.

2. Pumpanen J, Kolari P, Ilvesniemi H, Minkkinen K, Vesala T, et al. (2004) Comparison of different chamber techniques for measuring soil CO_2_ efflux. Agricultural and Forest Meteorology 123: 159-176.

3. Jaakkola A, Simojoki A (1998) Effect of soil wetness on air composition and nitrous oxide emission in a loam soil. Agricultural and Food Science 7: 491-505.

4. IPCC (2007) Climate Change 2007: Synthesis Report. Contribution of Working Groups I, II and III to the Fourth Assessment Report of the Intergovernmental Panel on Climate Change Geneva, Switzerland. 104 p.

5. Roslin T (2000) Dung beetle movements at two spatial scales. Oikos 91: 323-335.
