# Supplementary material for: Quantifying Beetle-Mediated Effects on Gas Fluxes from Dung Pats
Source: PLoS One. 2013 Aug 7;8(8):e71454. doi: 10.1371/journal.pone.0071454 (PMC3737124; doi:10.1371/journal.pone.0071454)
Supplement: Appendix S2 — Environmental conditions during the experiment. (DOC) [file pone.0071454.s002.doc]

**Appendix S2: Environmental conditions prevailing during the experiment**

As our experiment was conducted under a given set of environmental conditions, the patterns found are conditional on these circumstances. To allow the later comparison of our current findings with those exposed under other conditions, and to contribute to the exploration of effect sizes under variable environmental conditions, we here offer a description of the environmental circumstances prevailing during the experiment.

Environmental conditions were measured at two different scales: at the level of the dung pat and its immediate surroundings, and at the level of the Viikki Study and Research Farm:

At the scale of the dung pat, temperatures were recorded every hour throughout the duration of the experiment by a temperature logger (i-button, model DS1921G-F5#, Maxim Integrated, San Jose, CA, U.S.) inserted into a dung pat within a mesocosm (Fig. S1A). Ambient temperature in the free air space outside of the mesocosms was recorded on each of the measurement days by a digital thermometer (Fluke 51 II, Fluke, Norwich, UK) placed in the shade directly adjacent to each chamber (Fig. S1B). Overall loss of dung mass (reflecting both water loss and decomposition was recorded by weighing each pat at the start and end of the experiment (Fig. S1C).

At the level of the farm, average, maximum and minimum daily temperatures and precipitation was measured by an automatic weather transmitter (Vaisala WXT520, Vaisala Oy, Finland) located 540m m from the experimental field at a height of 10 m from the ground (Fig. S1D).

During the course of the experiment, the dung pats quickly dried out, as shown by rapid weight loss from dung pats both with and without beetles. On average, pats with beetles lost 95% of their weight, compared to 83% among pats without beetles (Fig. 1C). Major precipitation with a potential to impact the water-filled pore space (WFPS) occurred during a few days of the experiment (Fig. S1C). Overall, weather conditions during the summer of 2011 were close to, but slightly warmer than, the long-term average for 1971-2000, as measured by the Finnish Meteorological Institute . In Helsinki, June was approximately 2.5-3°C warmer and July 3.5-4°C warmer than the long-term average for 1971-2000.


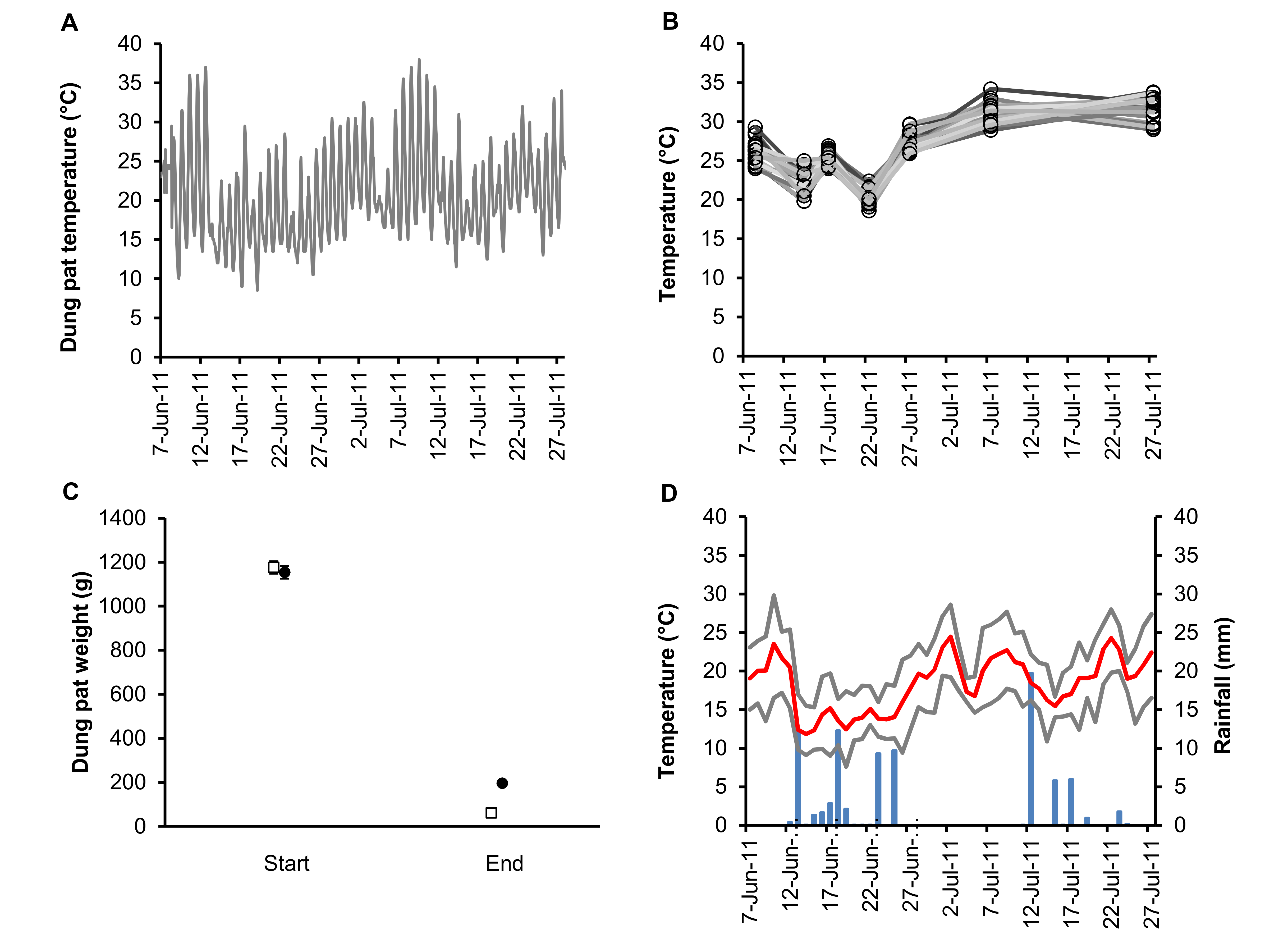


**Fig. S1. Environmental conditions during the course of the experiment of 2011.**
(A) Temperatures measured within an experimental dung pat. (B) Ambient temperature in the free air space outside of the mesocosms (with individual mesocosms identified by different shades of grey). (C) Dung mass (mean±SD of fresh weight) at the start and end of the experiment in pats with and without beetles (shown by white squares and black circles, respectively; SD too small to be distinguished for most data points). (D) Temperature (daily average shown by a red line, with maximum and minimum temperatures identified by grey lines) and precipitation (blue bars) measured by an automatic weather station operated 540m from the experimental field. In each panel, the range of the abscissa matches the start and end date of the experiment (June 7-27, 2011).

**References**
